# Practical Notes and Formulæ

**Published:** 1884-10-20

**Authors:** 


### PRACTICAL NOTES AND FORMULÆ.

**Thielemann's Cholera Mixture.**—The Druggists' Circular says: A preparation termed "Professor Thielemann's Cholera Mixture" has been very extensively employed in the northern countries of Europe, both by medical practitioners and the public at large, and has gained quite a reputation as a remedy against cholera morbus, diarrhœa, etc. In this country the compound has of late gained a foothold, and there seems to be a speedily growing demand for it from the drug stores and pharmacies especially in the parts of the country where the Scandinavian population has located. In the seventh edition of the Sweedish Pharmacopœia the compound was introduced under the synonym "Mixtura Thielemanni," and its composition is as follows:

MISTURA THIELEMANNI.

By weight.

| | | |
|---|---|---|
| ℞ | Oil of peppermint | 3 parts. |
| | Alcohol | 22 " |
| | Wine of opium and saffron | 10 " |
| | Wine of ipecac | 25 " |
| | Ethereal tincture of valerian | 40 " |

The same pharmacopœia directs the three last named ingredients to be prepared in the following manner:

WINE OF OPIUM AND SAFFRON.

| | | |
|---|---|---|
| ℞ | Ceylon cinnamon, in coarse powder | 1 part. |
| | Cloves, bruised | 1 " |
| | Spanish saffron | 5 parts |
| | Powdered opium | 15 " |
| | Malaga wine, sufficient to make | 150 " |

Macerate five days, under a temperature of 15-25° C. (59-77°F.), express and filter.

The wine of ipecac shall be prepared in the same manner, with one part of powdered ipecac and ten parts of sherry wine, and the ethereal tincture of valerian, with one part of valerian root in coarse powder, and five parts of spirit of ether (one part of ether and three parts of alcohol). The parts are all by weight. The pharmacopœial name for this tincture is Tinctura Valerianæ.

Dose, one to two teaspoonsful.

**Esmarch's Painless Caustic.**—

| | | |
|---|---|---|
| ℞ | Arsenious acid | 1 part. |
| | Sulphate of morphine | 1 " |
| | Calomel | 8 parts. |
| | Pulverized gum-Arabic | 48 " |

When used for warts, tumors, etc., the surface skin should be removed with a knife or blister, and the powder sprinkled on the denuded surface daily.—*St. Louis Druggist.*

**Formula for S. S. S.**—

℞ Old man's gray-beard root (Chionanthus Virginica)........................... 1 bushel.
Prickly ash root..........................16 ounces.
White and red sumac root, of each......... 8 "
Sarsaparilla root..........................10 "
Sulphate of copper......................... 8 drachms.

Sig.—One wineglassful four times a day. Strictly abstain from horseback riding, butter or very greasy food, all kinds of spirits or fermented liquors. Of course the chancre must be treated in the usual manner.

Bruise the gray-beard and sumacs roots and put them, with the sarsaparilla, in an iron pot sufficient to hold eight gallons of water, or cover the roots completely with the water. Cover the pot with pine tops and boil slowly until the liquid assumes the color of ink. Strain while warm, add the sulphate of copper and good Holland gin q. s. to prevent fermentation.

**Thompson's Eye Water.**—

℞ Sulphate of copper.......................10 grains.
Sulphate of zinc...........................40 "
Rose water................................. 2 pints.
Tincture of saffron......................... 4 drachms.
Tincture of camphor....................... 4 "

Mix and filter.

**Pulv. Anti-Chol. Infanti.**—The following prescription, sent to us by P. W. Burdge, M. D., of Rahway, N. J., is used at the Newark (N. J.) City Dispensary for cholera infantum :

R Hydrarg. sub mur............................ gr. ij
Pulv. ipecac..................................gr. iij
" piumbi acet.............................gr. viij
" Doveri...................................gr. vj
" creta comp...............................℈ ij.

Mice, et ft. chart. No. xxiv.—*Med. World.*

**For Piles.**—The following ointment will cure most cases of piles, even those that would seem to require an operation. A number of cases of chronic prolapsus ani have yielded to it :

℞ Monselle's salt............................ ʒ ss
Morphia sulph...............................grs. vj
Petroleum mass............................. ℥ j.

M.—Apply night and morning with finger.

Be sure to use petroleum mass ; lard, vaseline, cosmoline, etc., should not be substituted, as the crude petroleum has a specific effect on the parts diseased.—*Ibid.*

**Emmenagogue.**—The pill used by the French women to produce barrenness I have found a valuable emmenagogue. Each pill contains two grains each of aloes and carbonate of titanium. They have never failed to bring on the menstrual discharge at the next epoch. Stoppage produced by cold, etc., is restored by this preparation in eight hours. It is a most powerful direct stimulant to the sexual organs. I have no doubt that it would produce barrenness if persevered in. A married woman using the pills does not become pregnant.

My usual formula is as follows :

℞ Pulv. aloes (soc.) }
Carb. titan. } aa.......................... ʒ j.

Fiant pilulæ No. xxx. Sig.—One pill three times daily.

No particular nicety need be paid in regard to dose ; from one to three pills may be taken *ter die*. They should be commenced from one week to ten days before the expected menstrual period. I have employed them in many cases of amenorrhœa, both in retention and suppression, and almost invariably with the utmost gratifying results ; so certain are they to restore the uterine secretion, when suppression does not depend upon organic disease, that I almost regard them as a specific. Their action is peculiar ; they seem to possess the power of restoring the secretion when suppressed, and of promoting it when deficient. This pill is, in fact, a "female regulator." When the obstruction is from cold these pills, with warm pediluvia, are sufficient. They operate kindly and without excitement ; the patient hardly knows she is restored. —*Med. World.*

**Red color for Show Globes.**—In answer to the question, "How can a bright red color for show globes, that will not fade, be made ?" I offer the following :

℞ Carmine....................................grs. 1

Aq. amm.................................... ℥ 1

Glycerine.................................... ℥ 2

Aq. dest. q. s. ad.......................... ℥12

Dissolve the carmine in the ammonia. Mix all, and a beautiful and permanent red will be the result.—PHARMACIST.—*National Druggist.*

**Hill's Balsam of Honey and Tar.**—

℞ Balsam tolu.................................℥ 2

Styrax..........................................ʒ 2

Opium.......................................grns. 30

Honey..........................................℥ 8

Tar, pine tree.................................℥ 1

Alcohol........................................℥32

Mix and macerate for five days, with occasional agitation, then strain and bottle.—*National Druggist.*

